# Prognostic value of SYNTAX score, intravascular ultrasound and near-infrared spectroscopy in coronary disease: 12-year follow-up of ATHEROREMO

**DOI:** 10.1007/s00392-025-02756-8

**Published:** 2025-10-13

**Authors:** Victor G. Meuleman, Alexander Vanmaele, Jose M. Da Veiga Fernandes de Mira, K. Martijn Akkerhuis, Rohit M. Oemrawsingh, Maxime M. Vroegindewey, Jin M. Cheng, Hector M. Garcia-Garcia, Joost Daemen, Nicolas M. van Mieghem, Patrick W. Serruys, Robert Jan van Geuns, Eric Boersma, Isabella Kardys

**Affiliations:** 1 Department of Cardiology, Thorax Center, Cardiovascular Institute, Erasmus MC, P.O. Box 2040, Rotterdam, 3000 CA The Netherlands; 2 Bravis Hospital, Bergen Op Zoom & Roosendaal, The Netherlands; 3 Amphia Hospital, Breda, The Netherlands; 4 Department of Anesthesiology, Cardiovascular Institute, Erasmus MC, Rotterdam, The Netherlands; 5 Albert Schweitzer Hospital, Dordrecht, The Netherlands; 6 Interventional Cardiology, MedStar Washington Hospital Center, Washington, DC USA; 7 CORRIB Research Centre for Advanced Imaging and Core Laboratory, University of Galway, Galway, Ireland; 8 Radboud UMC, Nijmegen, The Netherlands

**Keywords:** Atherosclerosis, Intravascular ultrasound, Near-infrared spectroscopy, SYNTAX, Prognosis

## Abstract

**Background and aims:**

This study aims to investigate the very long-term predictive value of the SYNTAX score and plaque characteristics derived by intravascular ultrasound (IVUS) as well as near-infrared spectroscopy (NIRS), for all-cause mortality in patients with low to intermediate complex coronary artery disease.

**Methods:**

We evaluated 581 patients with chronic or acute coronary syndrome from the European Collaborative Project on Inflammation and Vascular Wall Remodeling in Atherosclerosis (ATHEROREMO) cohort. Flow-limiting lesions were treated with intracoronary stenting. IVUS-VH (*n* = 581) and NIRS (*n* = 195) images were obtained in a non-culprit segment ≥ 40 mm. Cox models were applied to relate pre-PCI SYNTAX score and plaque characteristics to very long-term all-cause mortality. Adjusted hazard ratios (aHR), corrected for cardiovascular comorbidities and risk factors, are reported per doubling of the corresponding variable.

**Results:**

Mean (standard deviation) age was 62 (11) years; 76% were men; median SYNTAX score was 9.0 (25th–75th percentile 4.0–15.0). Median follow-up was 12.8 (25th–75th percentile 10.1–13.4) years, and 177 cases of all-cause mortality occurred. SYNTAX score (aHR 1.25, 95% confidence interval [CI] 1.05–1.48; *p* = 0.012) and percentage dense calcified plaque (aHR 1.21 [1.02–1.42]; *p* = 0.026) were associated with increased mortality risk, while fibrous plaque (aHR 0.54 [0.34–0.87]; *p* = 0.011) was related to reduced risk. NIRS features were not associated with very long-term mortality.

**Conclusion:**

Angiography-based SYNTAX score and IVUS-VH-defined fibrous and dense calcified plaque were related to 12-year mortality in patients with low-to-intermediate complex CAD.

**Graphical Abstract:**

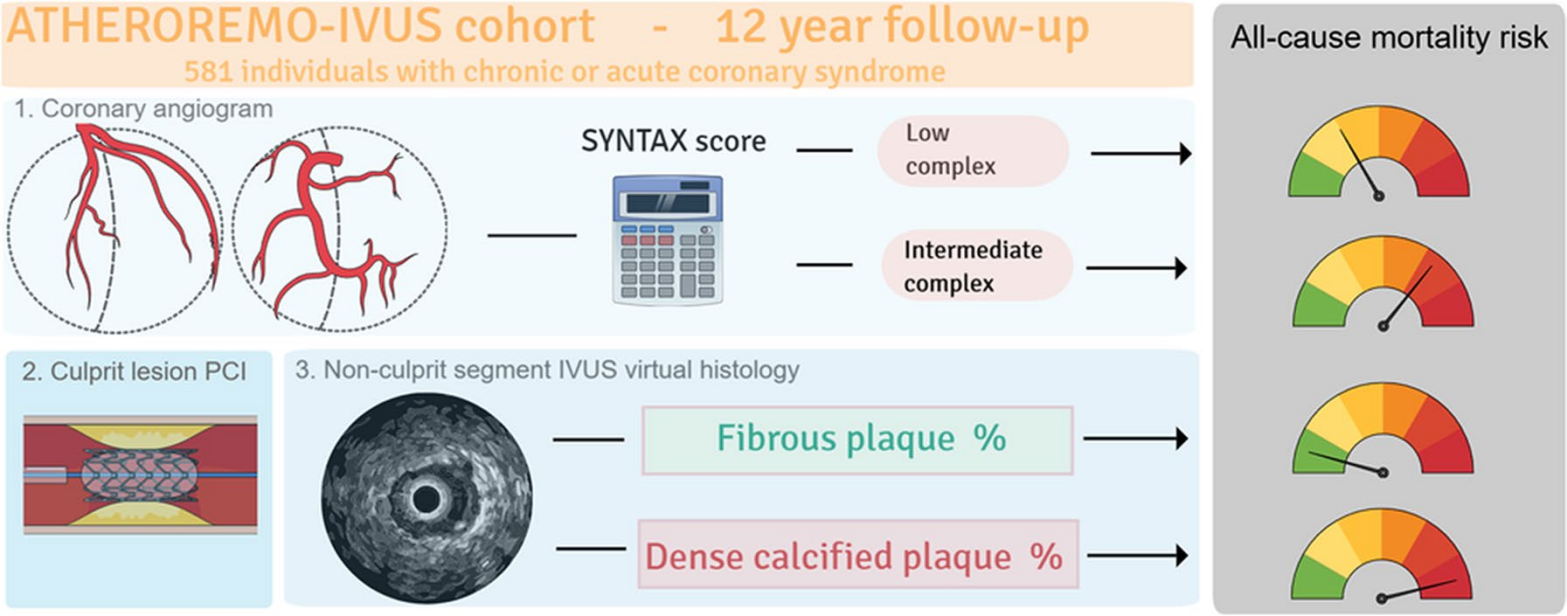

**Supplementary Information:**

The online version contains supplementary material available at 10.1007/s00392-025-02756-8.

## Introduction

Features derived from standard coronary angiography (CAG), as well as from sophisticated intracoronary imaging modalities, have proven to be of value for prognostication in patients with coronary artery disease (CAD) [1, 2]. The Synergy Between PCI With Taxus and Cardiac Surgery (SYNTAX) score is an angiographic tool to grade the severity and complexity of CAD, based on the anatomy of the complete coronary artery tree [3, 4]. It has been demonstrated to carry prognostic value for major adverse cardiac events (MACE) and all-cause death in patients with extensive CAD [4, 5]. In our previous investigations within the European Collaborative Project on Inflammation and Vascular Wall Remodeling in Atherosclerosis (ATHEROREMO) study, we have demonstrated the prognostic value of the SYNTAX score in patients with less complex CAD [6].

Intravascular ultrasound virtual histology (IVUS-VH) and near-infrared spectroscopy (NIRS) enable in vivo assessment of coronary plaque characteristics in CAD patients. Both techniques allow for differentiation of various atherosclerotic plaque phenotypes, which may in part determine patient outcome. Previously, in ATHEROREMO, we have demonstrated that the presence of a thin cap fibroatheroma (TCFA), large plaque burden, and small luminal area as assessed by IVUS-VH, as well as high lipid core burden index (LCBI) as assessed by NIRS, carry significant prognostic value for MACE at 1-year follow-up [7, 8]. These results remained consistent at 5 years of follow-up, although the prognostic relevance of TCFA was less evident [9, 10]. The fact that up to 75% of TCFAs may heal at 12 months follow-up, as demonstrated in an earlier study performing serial intracoronary imaging with IVUS-VH [11], may have contributed to these findings.

The value of the SYNTAX score, IVUS-VH, and NIRS for mortality prediction beyond 5 years has not yet been established in patients with low to intermediate complex CAD, and the current study aims to fill this gap. We report on the relation between the three invasive imaging instruments and 12-year all-cause mortality, as observed in the CAD patients who compose the ATHEROREMO cohort.

## Methods

### Study design and population

Design and detailed methods of the ATHEROREMO-IVUS study and ATHEROREMO-NIRS sub-study have been published previously [12]. In short, the study was conducted between 2008 and 2011 at Erasmus MC, Rotterdam, the Netherlands. A total of 581 patients with an indication for CAG due to chronic coronary syndrome (CCS; *N* = 254) or acute coronary syndrome (ACS; *N* = 323) were included. The clinical presentation of CAD was defined as CCS for stable patients (without myocardial infarction) or as ACS for unstable patients (including unstable angina pectoris and myocardial infarction). Upfront and when applicable, hemodynamically flow-limiting lesions in a culprit vessel were treated with stent implantation (Percutaneous Coronary Intervention [PCI]) according to the prevailing European Society of Cardiology guidelines. IVUS-VH and NIRS images were retrieved from a non-culprit coronary vessel and analyzed off-line by an independent core laboratory (Cardialysis, Rotterdam, the Netherlands) that was blinded for patient characteristics and outcome. Patient follow-up started at the day of study inclusion, the day the invasive coronary procedure was performed. The study was approved by the local Medical Ethics Committee and performed in accordance with the Declaration of Helsinki (REF). Written informed consent was obtained from all participants, which included approval for long-term follow-up.

### SYNTAX score

As recorded on the CAG (pre-PCI), all coronary lesions with more than 50% stenosis in vessels with a diameter of more than 1.5 mm were graded for severity and complexity, using a pre-specified point accreditation system, of which the sum resulted in the anatomical SYNTAX score [3]. A low score is defined as ≤ 22, an intermediate score as 23 to 32, and ≥ 33 as high score [5]. Higher scores indicate more complex coronary disease.

### IVUS imaging

After patient treatment was completed, based on the standard CAG, a proximal non-stenotic segment of a non-culprit coronary artery was selected for further invasive imaging according to a predefined study protocol [12] with the order of preference being the following: (1) left anterior descending; (2) right coronary artery; (3) left circumflex artery. The target segment was required to be at least 40 mm in length and without stenosis of more than 50%. IVUS images of this study segment were acquired with the Volcano Eagle Eye Gold IVUS 20 MHz catheter and analyzed with pcVH 2.1 and qVH software (Volcano Corp., San Diego, CA, USA). The extent of the segmental atherosclerotic plaques was assessed in the IVUS-VH-derived dataset. The external elastic membrane and luminal borders were contoured for each frame with a median interslice distance of 0.40 mm. Plaque burden (PB) was defined as the plaque and media cross-sectional area divided by the external elastic membrane cross-sectional area. A coronary lesion was defined as a segment with a plaque burden of more than 40% in at least three consecutive frames.

Analysis with IVUS-VH, which allows for in vivo differentiation of various plaque phenotypes, was used to classify four compositional features of segmental plaques: (1) fibrous: areas of densely packed collagen; (2) fibro-fatty: fibrous tissue with significant lipid interspersed in collagen; (3) necrotic core: necrotic regions consisting of cholesterol clefts, foam cells, and microcalcifications; (4) dense calcified: calcium depositing without adjacent necrosis [13, 14]. Additionally, three lesion types were scored [15]: (1) TCFA lesion, defined as a lesion with the presence of > 10% confluent necrotic core in direct contact with the lumen; (2) lesion with a plaque burden ≥ 70%; (3) lesion with a minimal luminal area (MLA) < 4.0mm^2^. Cutoff values of all measurements have been validated in other studies [1, 2, 7, 9, 16].

### NIRS imaging

NIRS imaging, which is capable of quantifying lipid accumulation in coronary plaques [17], was performed in the same study segment in which IVUS imaging was performed [12]. Images were acquired using a 3.2-F rapid exchange catheter, a pullback rotation device, and a console (InfraRedx, Burlington, Massachusetts). The NIRS catheter was automatically pulled back at a speed of 0.5 mm/s and 240 rotations per minute in a proximal segment of the non-culprit artery. Due to later availability of this catheter in our cathlab, a subset of patients was imaged with NIRS.

Based on the differential absorbance and scattering of near-infrared light at different wavelengths for different types of tissue, the lipid core burden (LCBI) can be measured[18]. A virtual color image was constructed for each pullback with the NIRS-catheter. This so-called chemogram maps the presence of lipid accumulation in the vessel wall, with red color representing low probability of lipid and yellow representing higher probability of lipid.

The fraction of yellow pixels obtained from the chemogram was multiplied by 1000 to compute the LCBI [18]. The following three measurements of the LCBI were quantified: (1) MaxLCBI_4mm_, the 4 mm segment with the maximum LCBI; (2) MaxLCBI_10mm_, the 10 mm segment with the maximum LCBI; (3) LCBI_ROI_, the region of interest of the investigated segment [17]. Cutoffs have been validated in other large studies [1, 2, 17, 19].

### Follow-up and endpoints

Follow-up started at inclusion and lasted for over 12 years. The study endpoint was all-cause mortality. Survival status was obtained from municipal civil registries in June 2023. Patients lost to follow-up were considered to be at risk until the date of last contact and censored thereafter.

### Statistical analysis

Distributions of continuous variables were tested for normality by the Kolmogorov–Smirnov test. Normally distributed continuous variables were presented as means with standard deviations, non-normally distributed continuous variables as medians and 25th–75th percentiles. Categorical variables were presented as numbers and percentages. IVUS determined volumes were normalized accordingly to segment length by dividing the volumes by their segment length and subsequently multiplying by the median (cohort) segment length.

Associations of SYNTAX score and IVUS and NIRS features with risk of mortality were investigated with Cox proportional hazard (PH) models. First, we used unadjusted models. Subsequently, based on existing literature, we adjusted for age, sex, hypertension, diabetes mellitus, dyslipidemia, renal impairment, history of myocardial infarction, history of PCI, history of CABG, history of peripheral artery disease, angiography indication (presentation at baseline with CCS or ACS), number of stenotic coronary vessels, index PCI performed, and statin use. These co-variates were selected based on other large studies [1, 2, 9]. In two separate models, we merely adjusted for segmental plaque burden or SYNTAX score, to exclude both segmental, and total coronary atherosclerotic burden as a potential confounder in the risk prediction of all-cause mortality [20]. Continuous variables were 2log-transformed for the Cox models to improve interpretability. Hence, the reported hazard ratios (HR) correspond with a doubling of the variable at hand. Schoenfeld residuals evaluation of the PH assumption did not show violations. Additionally, we visualized cumulative mortality incidence according to quartiles of compositional features.

Crude analyses were repeated in the patients with CCS and those with ACS separately. Interaction terms were examined to investigate possible differential effects between the two subgroups.

The statistical analyses were done with R (version 4.3.1.). Two-sided *p*-values < 0.05 were considered statistically significant.

## Results

### Baseline characteristics

The ATHEROREMO cohort consists of 581 participants; their baseline characteristics are shown in Table 1. The patients were 62 ± 11 (mean ± standard deviation (SD)) years old, and 439 (75.4%) were men. Acute coronary syndrome was the index event in 318 (54.7%), while 254 (43.7%) patients had CCS. A total of 511 (88.0%) patients underwent a PCI of a culprit vessel, and 520 (89.7%) were on statin therapy at discharge. The subset of 195 (33.6%) patients that underwent NIRS had a similar age (63 ± 11 years), proportion male sex (72.3%), number of performed index PCI’s (88.7%), and number of participants (89.2%) on statins at discharge as the full cohort. The indication for coronary imaging was slightly different than the full cohort: 46.6% had ACS, 53.3% had CCS.

**Table 1:** Baseline characteristics

	IVUS-VH	NIRS
n	581	195
Clinical characteristics		
Age (mean (SD))	61.55 (11.34)	63.74 (11.01)
Sex (% men)	439 (75.4)	141 (72.3)
BMI (kg/m^2^, mean (SD))	27.36 (3.97)	27.74 (4.53)
Smoking (current, %)	309 (52.8)	114 (58.5)
Hypertension (%)	300 (51.6)	108 (55.4)
Diabetes (%)	99 (17.0)	40 (20.5)
COPD (%)	27 (4.6)	10 (5.1)
Peripheral artery disease (%)	36 (6.2)	11 (5.6)
Positive family history (%)	301 (51.9)	115 (59.3)
Heart failure (%)	19 (3.3)	8 (4.1)
Prior CVA (%)	26 (4.5)	6 (3.1)
Prior myocardial infarction (%)	184 (31.7)	75 (38.5)
Prior PCI (%)	186 (32.0)	74 (37.9)
Prior CABG (%)	18 (3.1)	5 (2.6)
Renal impairment (%)	32 (5.5)	11 (5.6)
CRP (mg/L, mean (SD))	6.65 (16.80)	7.01 (17.73)
Dyslipidemia (%)	321 (55.3)	109 (55.9)
Lp(a) (nmol/L, mean (SD))	67.38 (79.45)	60.54 (69.66)
Procedural characteristics		
Indication for angiography (%)		
CCS	254 (43.7)	104 (53.3)
ACS	318 (54.7)	91 (46.6)
Other	9 (1.5)	0 (0)
Vessel disease (%)		
One	308 (53.0)	102 (52.3)
Two	168 (28.9)	57 (29.2)
Three	105 (18.1)	36 (18.5)
PCI Performed (%)	511 (88.0)	173 (88.7)
IVUS Vessel (%)		
RCA	176 (30.3)	56 (29.3)
CX	195 (33.6)	69 (36.1)
LAD	210 (36.1)	66 (34.6)
NIRS Vessel (%)		
RCA	-	58 (29.7)
CX	-	69 (35.4)
LAD	-	68 (34.9)
Medication usage at discharge		
Aspirin (%)	556 (96.5)	190 (97.4)
Thienopyridines (%)	543 (94.3)	185 (94.9)
Betablockers (%)	441 (77.8)	151 (77.4)
ACE-inhibitors (%)	376 (65.7)	116 (59.5)
Angiotensin-2 blockers (%)	14 (2.7)	8 (4.1)
Calcium Antagonists (%)	110 (19.1)	45 (23.1)
Loop diuretics (%)	75 (13.0)	33 (16.9)
Statins (%)	515 (89.6)	174 (89.2)

*ACS* acute coronary syndrome

*BMI* body mass index

*CABG* coronary artery bypass grafting

*CCS* chronic coronary syndrome

*COPD* chronic obstructive pulmonary disease

*CRP* C-reactive protein

*CVA* cerebro vascular accident

*CX* circumflex artery

*IVUS-VH* intravascular ultrasound virtual histology,

*LAD* left anterior descendens.

*Lp(a)* Lipoprotein a

*NIRS* near-infrared spectroscopy

*Positive family history* positive family history for cardiovascular disease

*PCI* percutaneous coronary intervention

*RCA* right coronary artery

### Lesion and plaque characteristics

Baseline IVUS-VH-derived lesion and plaque characteristics of the studied non-culprit segments are presented in Table 2. Two hundred forty-two (41.7%) study participants had at least one TCFA, which was more frequently found in the ACS group (45.0%), than in the CCS group (37.8%). Segmental plaque burden was comparable in the full cohort and in the two subgroups. Fibrous plaque was the most prevalent of four plaque phenotypes, with a median percentage of 56.0%. The median percentage of necrotic core plaque was 21.4%. Fibro-fatty and dense calcified plaques were less commonly found with a median below ten percent. Subgroups showed similar patterns for plaque type percentage.

**Table 2:** Baseline IVUS-VH lesion features and plaque characteristics as assessed by IVUS-VH and NIRS

	***Full cohort (n*** = ***581)***	***CCS (n*** = ***254)***	***ACS (n*** = ***318)***
***Lesion feature(s) by IVUS-VH***	***n*** (%)	***n*** (%)	***n*** (%)
PB ≥ 50%	446 (76.8%)	198 (78.0%)	240 (75.5%)
PB ≥ 70%	124 (21.3%)	61 (24.0%)	59 (18.6%)
MLA < 2.5 mm^2^	30 (5.2%)	16 (6.3%)	14 (4.4%)
MLA < 4 mm^2^	183 (31.5%)	86 (33.9%)	93 (29.2%)
TCFA	242 (41.7%)	96 (37.8%)	143 (45.0%)
TCFA & PB ≥ 70%	69 (11.9%)	36 (14.2%)	32 (10.1%)
TCFA & MLA < 4 mm^2^	61 (10.5%)	27 (10.6%)	33 (10.4%)
TCFA & PB ≥ 70% and MLA < 4 mm^2^	35 (6.0%)	19 (7.5%)	16 (5.0%)
***Plaque type by IVUS-VH***	**Median (Q**_**1**_**, Q**_**3**_**)**	**Median (Q**_**1**_**, Q**_**3**_**)**	**Median (Q**_**1**_**, Q**_**3**_**)**
Segmental Plaque volume (mm^3^)	222.5 (147.5, 325.7)	234.1 (150.9, 345.1)	215.1 (142.4, 304.9)
Segmental Plaque burden (%)	39.1 (30.0, 46.4)	40.1 (32.1, 47.4)	37.1 (28.4, 45.4)
Fibrous plaque percentage	58.1 (50.5, 65.8)	56.6 (49.5, 63.6)	59.8 (51.2, 66.4)
Fibrous plaques volume (mm^3^)	56.0 (27.0, 95.6)	58.8 (29.2, 105.7)	53.3 (23.4, 89.4)
Fibro-fatty plaque percentage	8.9 (5.7, 12.6)	9.4 (6.2, 13.4)	8.6 (5.3, 12.0)
Fibro-fatty plaque volume (mm^3^)	7.8 (3.3, 17.4)	9.2 (4.3, 20.8)	6.4 (2.9, 15.2)
Necrotic core plaque percentage	21.4 (16.9, 26.4)	21.5 (17.3, 25.3)	21.2 (16.4, 27.3)
Necrotic core plaque volume (mm^3^)	21.2 (8.5, 40.8)	21.0 (9.3, 43.7)	20.9 (8.0, 38.1)
Dense calcium plaque percentage	9.3 (5.1, 15.1)	10.9 (5.9, 16.6)	8.2 (4.9, 13.3)
Dense calcium plaque volume (mm^3^)	8.9 (2.9, 20.8)	10.4 (3.8, 23.1)	7.6 (2.2, 18.1)
***Plaque feature by NIRS***	**Median (Q**_**1**_**, Q**_**3**_**)**	**Median (Q**_**1**_**, Q**_**3**_**)**	**Median (Q**_**1**_**, Q**_**3**_**)**
LCBI region of interest (units)	41 (15, 82.5)	35 (14, 80.25)	46 (16, 86.5)
MaxLCBI4mm (units)	233.5 (93, 377)	201 (86.5, 377)	267 (105.5, 379.5)
MaxLCBI10mm (units)	131 (60.5, 245)	121 (49, 238)	153 (68.5, 249)

*ACS* acute coronary syndrome,

*CCS* chronic coronary syndrome,

*IVUS-VH* intravascular ultrasound virtual histology,

*LCBI* lipid core burden index,

*MaxLCBI4mm* 4 mm segment with the maximum amount of lipid core burden index,

*MaxLCBI10mm* 10 mm segment with the maximum amount of lipid core burden index,

*Median (Q*_*1*_*, Q*_*3*_*)* Median or 50th percentile (25th, 75th percentile),

*MLA* minimal luminal area,

*n* (%) study participants with at least one such lesion in their study segment,

*NIRS* near-infrared spectroscopy,

*PB* plaque burden,

*TCFA* thin cap fibro atheroma

In the subset of patients that underwent NIRS, the median LCBI values were slightly higher in the de ACS group compared to the CCS group (Table 2).

### Incidence of the study endpoint

Median (25th–75th percentile) follow-up time was 12.8 (10.1–13.4) years. During this period, 177 (30% of 581) cases of all-cause mortality occurred, of which 73 occurred in the NIRS subset (37% of 195).

### Association between the SYNTAX score and all-cause mortality

Median (25th–75th percentile) SYNTAX score of all study participants was 9.0 (4.0–15.0), of which 91.4% had a low, 7.9% had an intermediate, and less than 1% had a high SYNTAX score. After 12 years follow-up, the SYNTAX score was significantly associated with all-cause mortality, resulting in an adjusted hazard ratio (aHR) of 1.25 (95% confidence interval [CI] 1.05–1.48; *p* = 0.012) per doubling of the SYNTAX score (Table 3).

**Table 3:** IVUS-VH and NIRS features, and SYNTAX score, in relation to all-cause mortality

	*Unadjusted model*			*Adjusted model*		
***Lesion feature(s) by IVUS-VH***	**HR**	**95% CI**	***p*-value**	**HR**	**95% CI**	***p*-value**
PB ≥ 70%	1.27	0.90, 1.79	0.2	-	-	-
MLA < 4 mm^2^	1.07	0.78, 1.46	0.7	-	-	-
TCFA	0.84	0.61, 1.14	0.3	-	-	-
TCFA and PB ≥ 70%	1.19	0.77, 1.84	0.4	-	-	-
TCFA and MLA < 4 mm^2^	0.78	0.47, 1.31	0.4	-	-	-
TCFA and PB ≥ 70% and MLA < 4 mm^2^	0.99	0.54, 1.83	> 0.9	-	-	-
***Plaque type by IVUS-VH***	**HR**	**95% CI**	***p*-value**	**HR**	**95% CI**	***p*-value**
Segmental plaque volume	1.29	1.08, 1.55	0.006 *	1.14	0.94, 1.38	0.2
Segmental plaque burden	1.86	1.32, 2.64	< 0.001*	1.31	0.91, 1,89	0.2
Fibrous plaque percentage	0.44	0.29, 0.67	< 0.001 *	0.54	0.34, 0.87	0.011*
Fibrous plaques volume	1.10	1.00, 1.21	0.049*	1.05	0.96, 1.16	0.3
Fibro-fatty plaque percentage	1.09	0.91, 1.30	0.3	-	-	-
Fibro-fatty plaques volume	1.14	1.03, 1.26	0.009*	1.08	0.97, 1.20	0.2
Necrotic core plaque percentage	1.21	0.97, 1.51	0.084	-	-	-
Necrotic core plaque volume	1.13	1.03, 1.23	0.011*	1.07	0.98, 1.18	0.15
Dense calcium plaque percentage	1.42	1.21, 1.65	< 0.001*	1.21	1.02, 1.42	0.026*
Dense calcium plaque volume	1.23	1.12, 1.35	< 0.001*	1.11	1.01, 1.22	0.036*
***Plaque feature by NIRS***	**HR**	**95% CI**	***p*-value**	**HR**	**95% CI**	***p*-value**
LCBI region of interest	0.94	0.84, 1.06	0.3	-	-	-
MaxLCBI4mm	1.04	0.92, 1.19	0.5	-	-	-
MaxLCBI10mm	0.98	0.87, 1.11	0.8	-	-	-
	**HR**	**95% CI**	***p*-value**	**HR**	**95% CI**	***p*-value**
***SYNTAX score***	1.17	1.00, 1.37	0.047*	1.25	1.05, 1.48	0.012*

*Adjusted model* adjusted for age, sex, clinical risk factors, medical history, angiography indication, PCI performed, and statin use

*HR* hazard ratio. Continuous variables were log2 transformed; thus, estimates represent hazard ratios per doubling of these continuous variables

*IVUS-VH* intravascular ultrasound virtual histology

*LCBI* lipid core burden index

*MaxLCBI4mm* 4 mm segment with the maximum amount of lipid core burden index

*MaxLCBI10mm* 10 mm segment with the maximum amount of lipid core burden index

*MLA* minimal luminal area

*NIRS* near-infrared spectroscopy

*p*-value: *p < 0.05

*PB* plaque burden

*SYNTAX* Synergy Between PCI With Taxus and Cardiac Surgery

*TCFA* thin cap fibro atheroma

*95% CI* 95% confidence interval

#### Associations between IVUS and all-cause mortality

The percentage and volume of dense calcified plaques in the imaged non-culprit coronary segment was significantly associated with all-cause mortality. Respectively, a doubling in percentage and volume (mm^3^) entailed an aHR of 1.11 (95% CI 1.01–1.22; *p* = 0.036) and 1.21 (95% CI 1.02–1.42; *p* = 0.026) for mortality after correction for age, sex, and clinical characteristics (Table 3). After adjusting for segmental plaque burden and SYNTAX score, HRs for mortality were, respectively, 1.23 (95% CI 1.06–1.43; *p* = 0.008) and 1.19 (95% CI 1.06–1.33; *p* = 0.003) for dense calcified plaque volume, and 1.35 (95% CI 1.14–1.43; *p* < 0.001) and 1.19 (95% CI 1.06–1.33; *p* = 0.003) for percentage dense calcified plaque (Supplemental Table S1). A doubling in percentage segmental fibrous plaque was inversely associated with 12-year all-cause mortality (aHR 0.54, 95% CI 0.34–0.87; *p* = 0.011) (Table 3). After adjusting for segmental plaque burden and the SYNTAX score, HRs for percentage fibrous plaque in relation to mortality were, respectively, 0.52 (95% CI 0.33–0.83; *p* = 0.006) and 0.53 (95% CI 0.30, 0.96; *p* = 0.036) (Supplemental Table S1).

The relations of total segmental plaque volume, segmental plaque burden, and necrotic core plaque with all-cause mortality lost significance after adjustment for age, sex, and other clinical characteristics. We found no association between high-risk plaque features (TCFA, plaque burden ≥ 70% and MLA < 4mm^2^) and very long-term mortality. The results obtained from the Cox models using quartiles of compositional features were similar to those from analyses using continuous variables.

**Fig. 1:**
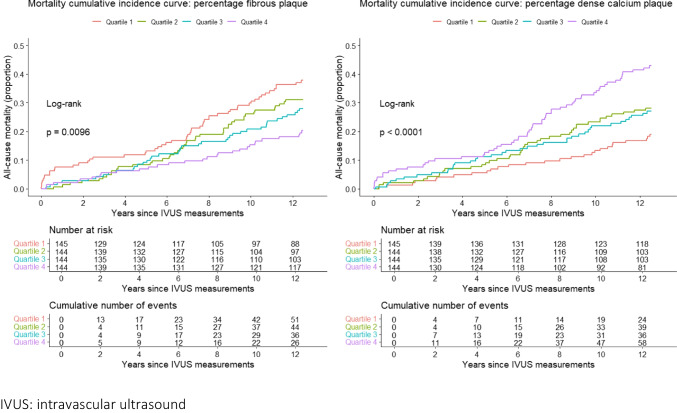
Mortality cumulative incidence by percentage fibrous plaque and dense calcified plaque

Cumulative mortality incidence categorized by quartiles of fibrous plaque percentage and dense calcium plaque percentage, as imaged by IVUS-VH, is shown in Fig. 1. Very long-term mortality of patients with high percentage dense calcified plaque showed to be significantly higher than for patients with low percentage of dense calcified plaque (*p*-value < 0.001). Higher percentage of fibrous plaque was associated with a lower mortality rate (*p*-value = 0.007).

#### Associations between NIRS and all-cause mortality

NIRS-derived plaque features were not associated with very long-term mortality; MaxLCBI_4mm_ with a HR of 1.04 (95% CI 0.92–1.19; *p* = 0.5), MaxLCBI_10mm_ with a HR of 0.98 (95% CI 0.87–1.11; *p* = 0.8), and LCBI_ROI_ with a HR of 0.94 (95% CI 0.84–1.06; *p* = 0.3), per doubling (Table 3).

#### Sensitivity analyses in CCS and ACS patients

The SYNTAX score was associated with 12-year all-cause mortality in the ACS subgroup, but not in the CCS subgroup. However, interaction terms were not statistically significant, and 95% CIs of the aHRs were largely overlapping. Hence, the evidence for a differential effect of the association between the SYNTAX score and mortality between the two subgroups is weak (Supplemental Table S2).

Sensitivity analyses further indicated that associations between segmental IVUS and NIRS plaque features and all-cause mortality found in the full cohort were presumably and primarily driven by the group that presented with ACS at baseline (Supplemental Table S2). However, again, analyses of interaction terms showed no evidence of differential effects between the CCS and ACS subgroups.

Cumulative survival per subgroup and percentage plaque type, as imaged by IVUS-VH, is presented in Supplemental Figs. S1 and S2.

#### Landmark analyses

The landmark analysis, with follow-up starting at 5 years, is presented in Supplemental Table S3. All plaque characteristics demonstrated similar associations with all-cause mortality in the landmark analysis, as in the analysis using full follow-up.

## Discussion

We investigated the value of the SYNTAX score and of coronary plaque characteristics, derived by IVUS-VH and NIRS, for 12-year prognostication. To the best of our knowledge, this is the first study that investigated such very long-term follow-up, and that investigated these relationships in patients with less extensive CAD, as reflected by the low to intermediate SYNTAX scores in more than 99% of our patients, which also included patients with myocardial infarction. We demonstrated that the SYNTAX score carries very long-term prognostic value in this patient population. The original SYNTAX studies included patients with stable advanced CAD, with mostly intermediate to high SYNTAX scores, but without myocardial infarction (3,4,5). Moreover, IVUS-VH-derived dense calcified plaque volume and percentage were associated with a higher risk of 12-year all-cause mortality, while a higher percentage of segmental fibrous plaque was associated with a lower risk of mortality. These findings remained consistent after adjusting for age, sex, and clinical characteristics, and after adjusting for segmental atherosclerotic burden or the SYNTAX score, a surrogate for multisegmental atherosclerotic burden.

Different plaque types co-exist in a single coronary segment, but the sequence of atherosclerotic lesion development has not been fully established. A previously proposed sequence of development starts with the formation of a fibrous plaque, gradually evolving into a necrotic core plaque, which may (repeatedly) rupture due to a thin fibrotic cap, heal, and gradually transform into a dense calcified plaque [21]. A greater calcium burden has been linked to lower levels of vascular inflammation and plaque vulnerability [22]. In this regard, a large calcium burden may be indicative of stable plaque. Nevertheless, dense calcified culprit lesions have been identified in ACS patients [23], and coronary calcification has been associated with worse post-PCI clinical outcomes including increased mortality [24], and with outcomes irrespective of treatment strategy (medical therapy only, PCI or CABG) [25]. In this context, dense calcified plaques could be indicative of more advanced CAD, which may entail adverse consequences. Our study results, demonstrating the prognostic value of fibrous and dense calcified plaques for 12-year mortality, support the hypothesis that fibrous plaques are more indicative of an early stage of CAD, and dense calcified plaques are indicative of more advanced CAD [21].

The extent of the atherosclerotic burden in the IVUS-VH-imaged non-culprit segment, as reflected by PB, plaque volume, lumen volume, and MLA, was not associated with mortality in this very long-term follow-up study. Segmental imaging was done in a single coronary artery, unlike the two to three vessel models used in the LRP and PROSPECT studies[1, 2, 16], which might have contributed to our findings. Examining a single coronary artery may seem profitable when hypothesizing that its features are representative of the entire coronary tree; however, multivessel intracoronary imaging still is likely to better estimate the full extent of atherosclerotic burden.

High-risk “vulnerable” plaque features like TCFA and high LCBI also were no longer associated with mortality at very long-term follow-up. Dynamic plaque morphology over time, with high-risk plaques becoming quiescent and stable plaques becoming obstructive as time progresses, may have contributed to these findings [26]. In a recent intracoronary imaging study, with serial OCT at baseline and six months, progression of calcification in plaques was associated with significant reduction in inflammatory features like TCFA [27]. Such features associated with plaque dynamics can pose additional challenges for long-term prognostication. Since only 195 patients underwent NIRS-imaging, it should be emphasized that larger studies are needed to confirm the lack of association between NIRS findings at baseline and long-term outcomes.

Finally, associations between the SYNTAX score and all-cause mortality showed no statistically significant differential effects between the CCS and ACS subgroups. Also, we found no statistically significant interaction between percentage fibrous and dense calcium plaques and the subgroups for the associations with mortality. Nevertheless, our exploratory subgroup analyses demonstrated that associations between fibrous and dense calcium percentage with all-cause mortality, found in the full cohort, were mainly driven by the group that presented with ACS at baseline; and in the latter group, necrotic core percentage was also found to be a potentially relevant predictor.

### Limitations

Several study limitations have to be acknowledged. First, our data were obtained in a single academic center, which may influence generalizability. Second, repeat intracoronary imaging with IVUS-VH and NIRS was not performed; hence, the long-term evolution of baseline plaque characteristics could not be evaluated. Third, follow-up information on the (change in) LDL levels and the use of cardiovascular medication, in particular statins and PCSK9 (Proprotein Convertase Subtilisin/Kexin type 9)-inhibitors was not registered. Fourth, we investigated anatomical and chemical coronary plaque features, as well as the extent of the atherosclerotic burden. In addition to that, the degree of metabolic activity and thrombogenic activation in a patient could have an important impact on the risk determination of CAD as well [28, 29]. These parameters were not investigated in our study. Fifth, plaque volume, PB, and MLA were derived from IVUS-VH images in our study, but it should be noted that contemporary greyscale IVUS provides easier access to these parameters. Finally, although using cardiac death as an outcome measure could have provided additional insights into the prognostic value of the studied plaque characteristics, cause of death data was not available for this investigation.

## Conclusion

The SYNTAX-score, derived from standard coronary angiography, and percentage segmental fibrous and dense calcified plaque, as imaged with IVUS-VH, predicts 12-year mortality risk in patients with low to intermediate complex coronary artery disease.

## Supplementary Information

Below is the link to the electronic supplementary material.

ESM 1 (DOCX 1.23 MB)

## Data Availability

Anonymized data that support the findings of this study will be made available to other researchers for purposes of reproducing the results upon reasonable request and in accordance with a data-sharing agreement.
