# Supplementary material for: Prognostic value of SYNTAX score, intravascular ultrasound and near-infrared spectroscopy in coronary disease: 12-year follow-up of ATHEROREMO
Source: Clin Res Cardiol. 2025 Oct 13;115(1):78–88. doi: 10.1007/s00392-025-02756-8 (PMC12783281; doi:10.1007/s00392-025-02756-8)
Supplement: Supplementary file 1 — (DOCX 1.23 MB) [file 392_2025_2756_MOESM1_ESM.docx]

**Supplemental table S1**: Prognostic IVUS-VH features, in relation to all-cause mortality in the full cohort, adjusted for segmental plaque burden, or adjusted for SYNTAX score

|  | ***Adjusted for segmental PB*** | | | ***Adjusted for SYNTAX score*** | | |
| --- | --- | --- | --- | --- | --- | --- |
| ***Plaque type by IVUS-VH*** | **HR** | **95% CI** | ***p*-value** | **HR** | **95% CI** | ***p*-value** |
| Fibrous plaque percentage | 0.52 | 0.33, 0.83 | 0.006* | 0.53 | 0.30, 0.96 | 0.036* |
| Dense calcium plaque percentage | 1.35 | 1.14, 1.59 | <0.001* | 1.34 | 1.11, 1.62 | 0.002* |
| Dense calcium plaque volume | 1.23 | 1.06, 1.43 | 0.008* | 1.19 | 1.06, 1,33 | 0.003* |

HR: Cox proportional hazard ratio

IVUS-VH: intravascular ultrasound virtual histology

PB: plaque burden

*p*-value: * *p* < 0.05

SYNTAX (Synergy Between PCI With Taxus and Cardiac Surgery)

95% CI: confidence interval

**Supplemental table S2.** Hazard ratios for all-cause mortality, in CCS and ACS patients, for IVUS-VH and NIRS features, and SYNTAX score.

|  | ***CCS group*** | | ***ACS group*** | | ***Differential effect*** |
| --- | --- | --- | --- | --- | --- |
| ***Lesion feature(s) by IVUS-VH*** | **HR** | **95% CI** | **HR** | **95% CI** | ***p*-value** |
| PB ≥ 70% | 0.89 | 0.54, 1.48 | 1.62 | 0.99, 2.65 | 0.1810 |
| MLA < 4mm^2^ | 1.21 | 0.78, 1.86 | 0.95 | 0.59, 1.53 | 0.4530 |
| TCFA | 1.06 | 0.67, 1.66 | 0.64 | 0.41, 0.98 | 0.2100 |
| TCFA & PB ≥ 70% | 0.92 | 0.49, 1.74 | 1.46 | 0.78, 2.75 | 0.4105 |
| TCFA & MLA4 < 4mm^2^ | 0.91 | 0.46, 1.82 | 0.73 | 0.34, 1.58 | 0.6710 |
| TCFA & PB ≥ 70% & MLA < 4mm^2^ | 0.87 | 0.38, 1.99 | 1.16 | 0.47, 2.85 | 0.6922 |
|  |  |  |  |  |  |
| ***Plaque type by IVUS-VH*** | **HR** | **95% CI** | **HR** | **95% CI** | ***p*-value** |
| Segmental plaque volume | 1.10 | 0.86, 1.41 | 1.35 | 1.04, 1.75 | 0.2118 |
| Segmental plaque burden | 1.23 | 0.74, 2.03 | 2.05 | 1.27, 3.31 | 0.1029 |
| Fibrous plaque percentage | 0.38 | 0.19, 0.77 | 0.47 | 0.27, 0.81 | 0.4649 |
| Fibrous plaques volume | 1.01 | 0.89, 1.14 | 1.13 | 0.99, 1.29 | 0.1778 |
| Fibro-fatty plaque percentage | 1.17 | 0.88, 1.54 | 0.96 | 0.76, 1.21 | 0.3290 |
| Fibro-fatty plaques volume | 1.05 | 0.91, 1.21 | 1.14 | 0.99, 1.31 | 0.3798 |
| Necrotic core plaque percentage | 1.04 | 0.78, 1.39 | 1.46 | 1.04, 2.04 | 0.1829 |
| Necrotic core plaque volume | 1.03 | 0.91, 1.17 | 1.17 | 1.03, 1.34 | 0.1437 |
| Dense calcium plaque percentage | 1.21 | 0.98, 1.50 | 1.68 | 1.33, 2.10 | 0.0993 |
| Dense calcium plaque volume | 1.12 | 0.98, 1.27 | 1.30 | 1.14, 1.49 | 0.1402 |
|  |  |  |  |  |  |
| ***Plaque feature by NIRS*** | **HR** | **95% CI** | **HR** | **95% CI** | ***p*-value** |
| LCBI region of interest | 0.93 | 0.78, 1.10 | 0.97 | 0.83, 1.13 | 0.8600 |
| MaxLCBI4mm | 1.01 | 0.83, 1.24 | 1.08 | 0.92, 1.27 | 0.7130 |
| MaxLCBI10mm | 0.97 | 0.82, 1.16 | 1.02 | 0.87, 1.20 | 0.8800 |
|  |  |  |  |  |  |
|  | **HR** | **95% CI** | **HR** | **95% CI** | ***p*-value** |
| ***SYNTAX score*** | 1.04 | 0.84, 1.28 | 1.40 | 1.11, 1.78 | 0.0794 |

ACS: acute coronary syndrome

CCS: chronic coronary syndrome

CI: confidence interval

HR: (unadjusted) hazard ratio

IVUS-VH: intravascular ultrasound virtual histology

LCBI: lipid core burden indexMaxLCBI4mm: 4mm segment with the maximum amount of lipid core burden index

MaxLCBI10mm: 10mm segment with the maximum amount of lipid core burden indexMLA: minimal luminal area

NIRS: near-infrared spectroscopy

*p*-value: *p* > 0.05: indicating no differential effects between CCS and ACS subgroups,

PB: plaque burden

SYNTAX (Synergy Between PCI With Taxus and Cardiac Surgery)

TCFA: thin cap fibro atheroma

**Supplemental table S3:** Five-year landmark analysis of IVUS-VH and NIRS features, in relation to all-cause mortality

|  | *Main analysis*  (unadjusted) | |  | *5-year landmark analysis (unadjusted)* | |  |
| --- | --- | --- | --- | --- | --- | --- |
| ***Lesion feature(s) by IVUS-VH*** | **HR** | **95% CI** | ***p*-value** | **HR** | **95% CI** | ***p*-value** |
| PB ≥ 70% | 1.27 | 0.90, 1.79 | 0.2 | 1.46 | 0.78, 1.80 | 0.4 |
| MLA < 4mm^2^ | 1.07 | 0.78, 1.46 | 0.7 | 1.47 | 0.76, 1.61 | 0.6 |
| TCFA | 0.84 | 0.61, 1.14 | 0.3 | 0.72 | 0.50, 1.05 | 0.09 |
| TCFA & PB ≥70% | 1.19 | 0.77, 1.84 | 0.4 | 1.03 | 0.59, 1.80 | 0.9 |
| TCFA & MLA4 < 4mm^2^ | 0.78 | 0.47, 1.31 | 0.4 | 0.84 | 0.46, 1.52 | 0.6 |
| TCFA & PB ≥ 70% & MLA < 4mm^2^ | 0.99 | 0.54, 1.83 | >0.9 | 1.04 | 0.51, 2.14 | 0.9 |
|  |  |  |  |  |  |  |
| ***Plaque type by IVUS-VH*** | **HR** | **95% CI** | ***p*-value** | **HR** | **95% CI** | ***p*-value** |
| Fibrous plaque percentage | 0.44 | 0.29, 0.67 | <0.001 * | 0.41 | 0.22, 0.77 | 0.005* |
| Fibro-fatty plaque percentage | 1.09 | 0.91, 1.30 | 0.3 | 1.14 | 0.86, 1.51 | 0.4 |
| Necrotic core plaque percentage | 1.21 | 0.97, 1.51 | 0.084 | 1.38 | 0.95, 2.01 | 0.087 |
| Dense calcium plaque percentage | 1.42 | 1.21, 1.65 | <0.001* | 1.57 | 1.23, 2.00 | <0.001* |
| Segmental plaque burden | 1.86 | 1.32, 2.64 | <0.001* | 1.98 | 0.50, 3.36 | 0.011* |
|  |  |  |  |  |  |  |
| ***Plaque feature by NIRS*** | **HR** | **95% CI** | ***p*-value** | **HR** | **95% CI** | ***p*-value** |
| LCBI region of interest | 0.94 | 0.84, 1.06 | 0.3 | 0.91 | 0.77, 1.07 | 0.4 |
| MaxLCBI4mm | 1.04 | 0.92, 1.19 | 0.5 | 1.00 | 0.84, 1.19 | 1.0 |
| MaxLCBI10mm | 0.98 | 0.87, 1.11 | 0.8 | 0.93 | 0.79, 1,09 | 0.4 |

HR: hazard ratio, continuous variables were log2 transformed, thus estimates represent hazard ratios per twofold increase of these continuous variables

IVUS-VH: intravascular ultrasound virtual histology

LCBI: lipid core burden index

Main analysis: follow-up starting at inclusion

MaxLCBI4mm: 4mm segment with the maximum amount of lipid core burden index

MaxLCBI10mm: 10mm segment with the maximum amount of lipid core burden index

MLA: minimal luminal area

NIRS: near-infrared spectroscopy

PB: plaque burden

*p*-value: * *p* < 0.05

TCFA: thin cap fibro atheroma

5-year landmark analysis: follow-up starting at the 5-year landmark

95% CI= 95% confidence interval

**Supplemental figure S1:** Mortality cumulative incidence according to plaque type by IVUS in the CCS group


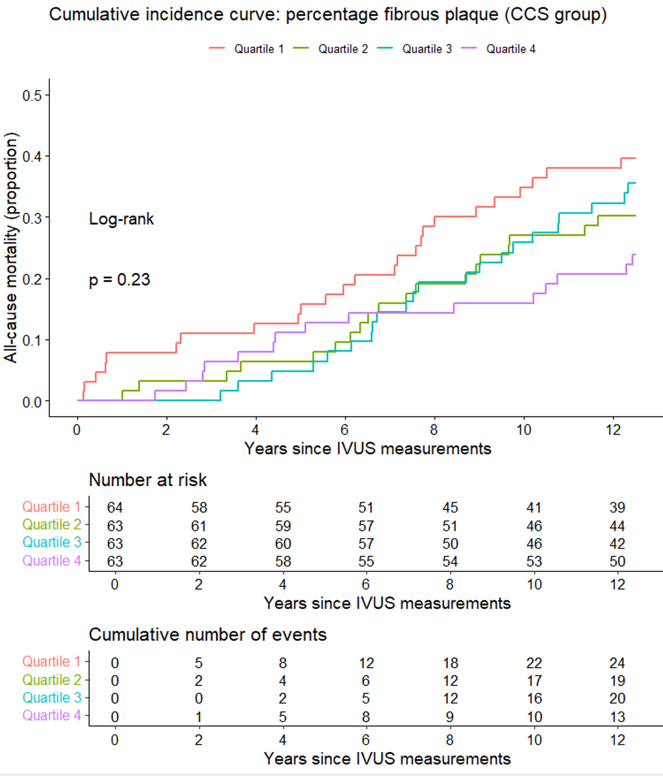


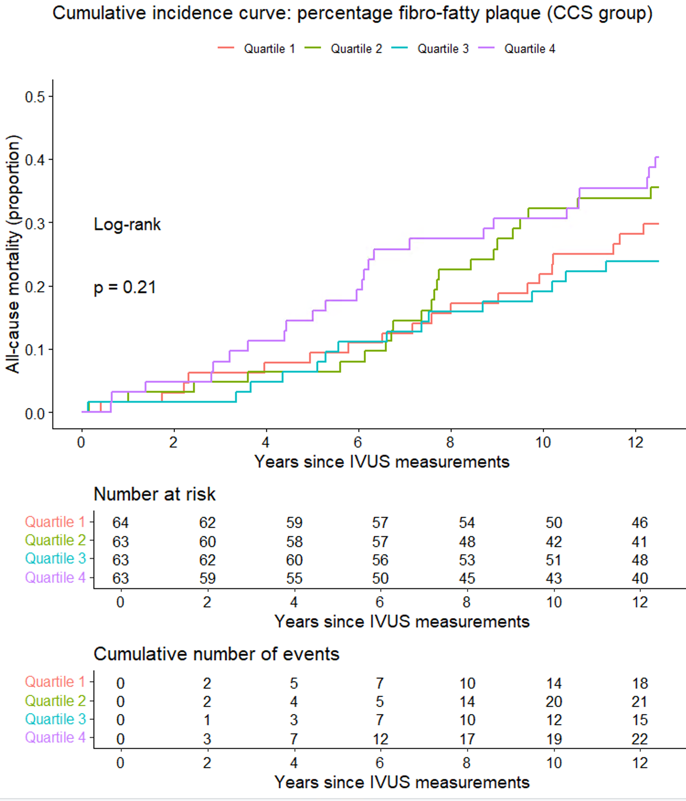


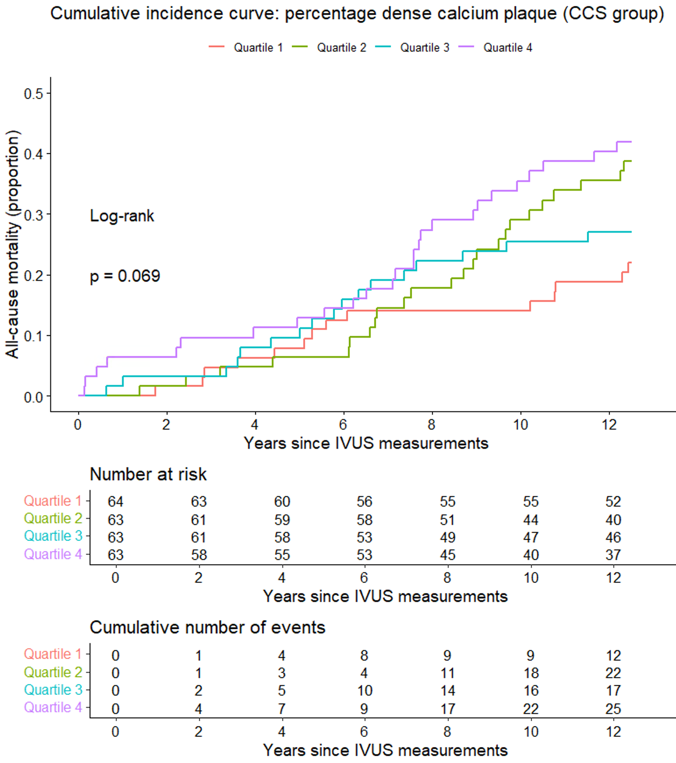

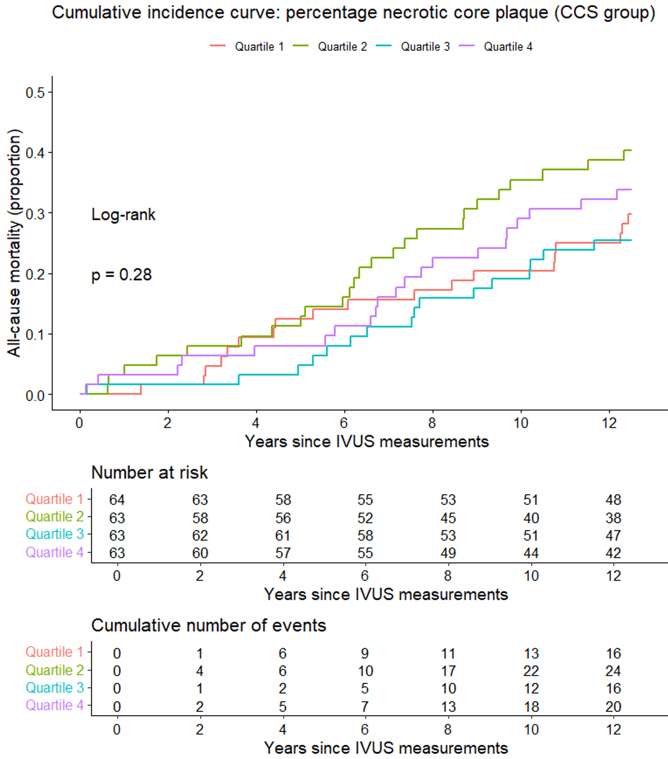


CCS: chronic coronary syndrome

IVUS: intravascular ultrasound

**Supplemental figure S2:** Mortality cumulative incidence according to plaque type by IVUS in the ACS group


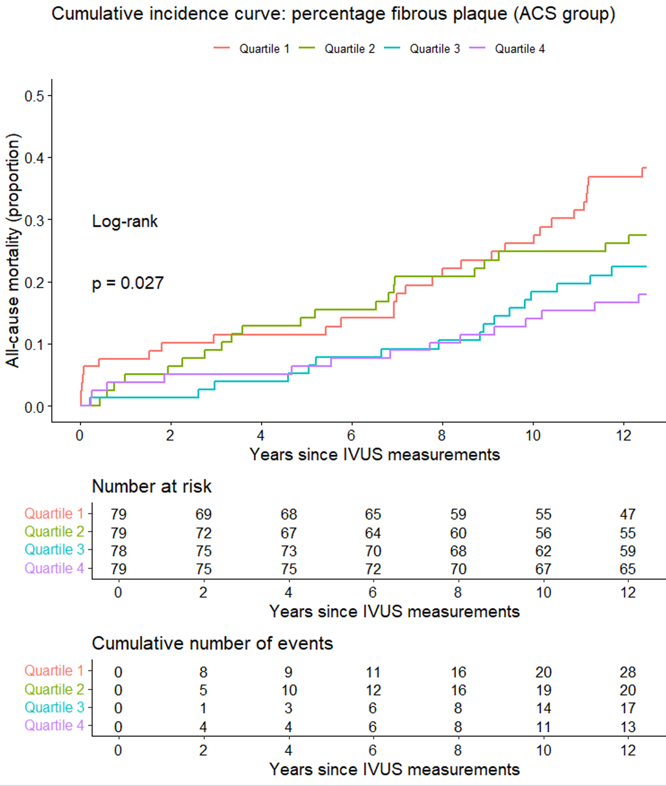

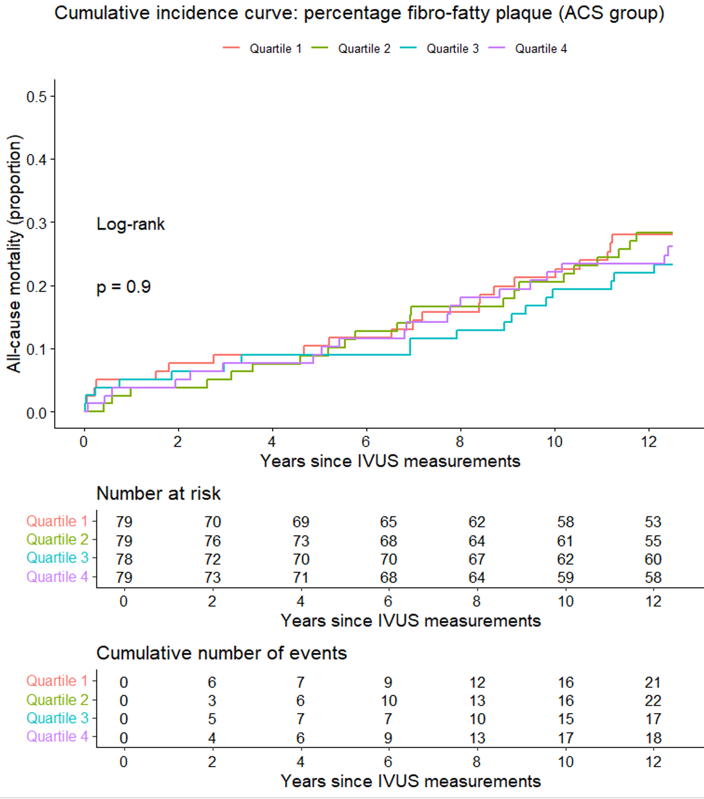


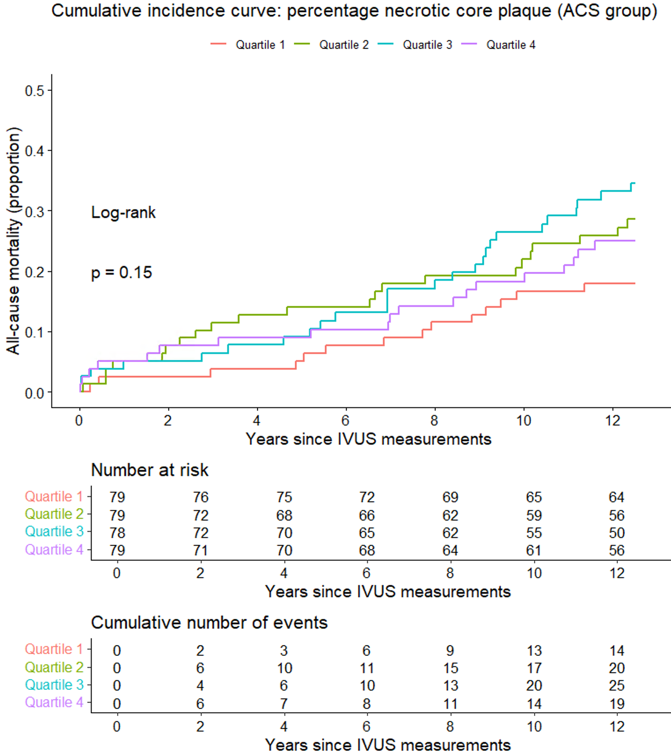


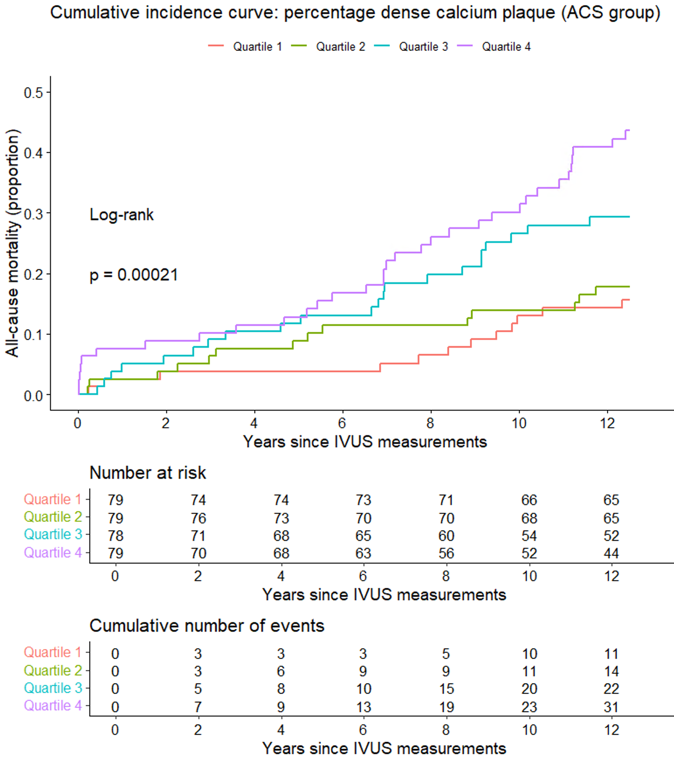


ACS: acute coronary syndrome

IVUS: intravascular ultrasound
